# Does smoking increase the anesthetic requirement?

**DOI:** 10.3906/sag-1602-57

**Published:** 2019-10-24

**Authors:** Erdoğan ÖZTÜRK, Mustafa Said AYDOĞAN, Kazım KARAASLAN, Zafer DOĞAN, Ufuk TOPUZ

**Affiliations:** 1 İstanbul Özel Memorial Hizmet Hospital, İstanbul Turkey; 2 Department of Anesthesiology and Reanimation, Faculty of Medicine, İnönü University, Malatya Turkey; 3 Department of Anesthesiology and Reanimation, Faculty of Medicine, Bezmialem Vakıf University, İstanbul Turkey; 4 Department of Anesthesiology and Reanimation, Faculty of Medicine, Biruni University, İstanbul Turkey; 5 Acıbadem Taksim Hospital, İstanbul, Turkey and Anesthesia Program,Health Vocational School, İstanbul Esenyurt University, İstanbul Turkey

**Keywords:** Smoking, environmental tobacco smoke, anesthetic agent consumption

## Abstract

**Background/aim:**

To examine the effects of active and passive smoking on perioperative anesthetic and analgesic consumption.

**Materials and methods:**

Patients were divided into three groups: group S, smokers; group PS, passive smokers; and group NS, individuals who did not have a history of smoking and were not exposed to smoke. All patients underwent the standard total intravenous anesthesia method. The primary endpoint of this study was determination of the total amount of propofol and remifentanil consumed.

**Results:**

The amount of propofol used in induction of anesthesia was significantly higher in group S compared to groups PS and NS. Moreover, the total consumption of propofol was significantly higher in group S compared to groups PS and NS. The total propofol consumption of group PS was significantly higher than that of group NS (P = 0.00). Analysis of total remifentanil consumption showed that remifentanil use was significantly higher in group S compared to group NS (P = 0.00).

**Conclusion:**

The amount of the anesthetic required to ensure equal anesthetic depth in similar surgeries was higher in active smokers and passive smokers compared to nonsmokers.

## 1. Introduction

Tobacco smoke consists of more than 4000 particles of toxic, ciliatoxic, and carcinogenic properties in gas and particle phases [1,2]. Nonsmokers exposed to secondhand smoke in their environments are described as passive smokers.

The risk for anesthesia-associated reintubation, laryngospasm, bronchospasm, aspiration, hypoventilation, and hypoxemia is 1.8 times greater in smokers compared to nonsmokers. This rate is 2.3 times higher in younger smokers and 6.3 times higher in obese smokers. In addition, the risk of developing bronchospasm is 25.7 times higher in female smokers than in male smokers [3].

Tobacco smoke induces hepatic microsomal enzymes and therefore increases the metabolism of drugs such as phenytoin, chlorpromazine, fentanyl, theophylline, and others. While it has been shown that the dose requirements for benzodiazepine increase in smokers, there has been no change reported in lidocaine and corticosteroids requirements [2]. In the literature there are limited studies investigating the anesthetic requirements in patients who smoke; however, we did not find any studies investigating the anesthetic requirements for passive smokers. In this study, we examined the effects of active and passive smoking on perioperative anesthetic and analgesic consumption.

## 2. Materials and methods

This study was approved by the ethics committee of İnönü University Faculty of Medicine and consisted of 90 adult patients with American Society of Anesthesiologists (ASA) I-II physical scores and who were scheduled for total abdominal hysterectomy at the department of obstetrics and gynecology.

**Figure 1:**
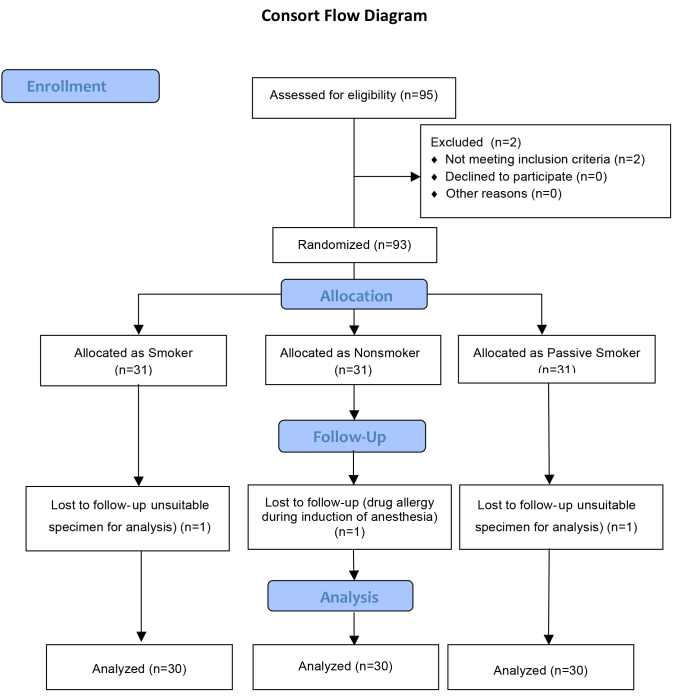
Consort flow diagram of patients.

Patients who did not consent to participate, patients with psychiatric problems, drug or alcohol abusers, patients who used drugs known to cause hypersensitivity, and patients with systolic arterial pressure greater than 160 mmHg and diastolic blood pressure greater than 90 mmHg or heart rates lower than 50 beats/min were excluded from the study (Figure 1).

Patients who fulfilled the criteria for inclusion in the study were interviewed regarding their history of smoking and the presence of smokers in their environments one day before the operation. Patient responses were placed in a sealed envelope and were not opened until the study was terminated. Active smoker patients with a history of smoking 10 cigarettes per day over a period of one year or longer were placed in group S; passive smoker patients exposed to cigarette smoke every day for at least one year through sharing their environment with people who smoked daily were placed in group PS; and patients without a history of smoking or exposure to smoke were placed in group NS. Various measurements, such as cotinine, carbon monoxide, and thiocyanate, can be taken to determine whether a person smokes. Since carbon monoxide and thiocyanate can be acquired through environmental sources, their measurement for the purpose of evaluating tobacco use may give misleading results. With active or passive cigarette smoking, nicotine is absorbed from the lungs and mucous membranes in the mouth and is immediately metabolized to cotinine in the body. The cotinine can be detected even several days after termination of smoking. Therefore, we planned to analyze the serum cotinine levels in the patients’ blood in order to eliminate self-report errors. Prior to starting the operation and after placing the intravenous catheter, a 3 mL blood sample was collected in order to analyze serum cotinine levels. The serum samples were stored at –80 °C until all samples were obtained; all samples were examined together. Serum cotinine levels were measured by the competitive micropallets immunoassay method using a commercially available cotinine kit (Cotinine EIA, Florence, Italy). Serum cotinine levels >50 ng/mL indicated that the patient was an active smoker; serum cotinine levels 11–50 ng/mL indicated that the patient had recently quit smoking or was a passive smoker; and serum cotinine levels 1–10 ng/mL indicated that the patient was a nonsmoker.

Preoperative premedication was not given to any patient. In the operating room all patients were closely monitored via ECG (DII), pulse oximetry (SpO2), noninvasive blood pressure, and body temperature. Then, a Ringer’s lactate infusion was started. The BIS monitor (A-2000 Bispectral Index, Aspect Medical Systems, the Netherlands) was used to assess the depth of anesthesia. Patient foreheads and temporal regions were cleaned with alcohol, and the BIS sensor (BIS Quatro, Aspect Medical Systems, the Netherlands) was placed.

The standard anesthesia technique was applied to all patients. Thirty seconds after a remifentanil infusion of 0.5 μg/kg/min dose was started, 0.5 mg/kg propofol bolus was applied. Every 20 s after the bolus dose was given a verbal warning was issued and an additional dose of 20 mg propofol was given until the response to stimulation disappeared. Following loss of consciousness, a 75 μg/ kg/min propofol infusion was started. Then, 0.6 mg/ kg atracurium was given, and respiratory support was provided for 3 min with a face mask. After BIS values ˂45 and adequate muscle relaxation were achieved, patients were intubated with an endotracheal tube. After intubation, all patients were continuously mechanically ventilated using a Dräger Cato edition (Dräger, Germany) anesthesia machine with a 40% O2–air mixture at intermittent positive pressure ventilation mode with a tidal volume (6–8 mL/ kg), a respiratory frequency of 10–12 min and end-tidal CO2 values 30–35 mmHg. The remifentanil infusion rate was reduced by 50%. The BIS value was kept between 45 and 60 throughout the surgery. BIS, mean arterial blood pressure (MAP), and heart rate (HR) values were measured and recorded at the start (t0), before intubation (t1), 5 min after intubation (t2), intraoperative 10 min (t3), 20 min (t4), 30 min (t5), 40 min (t6), 50 min (t7), 60 min (t8), and prior to extubation (t9).

An additional 20 mg propofol dose was administered, and the propofol infusion rate was increased by 50% when superficial signs of anesthesia (movement and facial grimacing) were observed or the BIS level was >60. The remifentanil infusion rate was arranged so that MAP and HR were ±20% of their starting values. In cases where hypertension or tachycardia occurred, administration of 1 μg/kg remifentanil bolus was planned; if the patient did not respond to that bolus dose after 1 min, an additional bolus dose would be administered. If hypotension developed, fluid therapy and a 50% reduction in the rate of infusion of remifentanil were planned. If, despite this treatment, hypotension could not be corrected, 5 mg of ephedrine would be administered.

All surgical operations were performed in a similar manner by the same surgical team. After closing the surgical field, the infusion of propofol and remifentanil was terminated, and patients were ventilated with 6 L/ min 100% O2. Then, after establishing that adequate spontaneous breathing and muscle strength were achieved, patients were extubated.

The primary endpoint of the study was determination of the total amount of propofol and remifentanil consumed, while the second endpoint was examination of perioperative MAP, HR, and BIS values.

## 3. Results

Demographic characteristics such as age, height, body weight, ASA, and duration of surgery and anesthesia for all patients are shown in Table 1. There were no statistically significant differences between groups in the comparison of these data (P > 0.05).

**Table 1:** Demographic characteristics and duration of anesthesia and surgery (values are presented as mean ± SD).

	Group Sn (%)	Group PSn (%)	Group NSn (%)
Low (1–10 ng/mL)	0 (0)	2 (6.6)	30 (100)
Moderate (11–50 ng/mL)	1 (3.3)	28 (93.3)	0 (0)
High (>50 ng/mL)	29 (96.6)	0 (0)	0 (0)

SD: Standard deviation ASA: American Society of Anesthesiologists score

The comparison of serum cotinine levels among groups showed that there was a statistically significant difference between groups (Table 2).

**Table 2:** Results of cotinine serum levels.

	Group S (n = 30)	Group PS(n = 30)	Group NS(n = 30)	P-value
Age (year)	45.38 ± 6.77	43.38 ± 11.45	46.63 ± 8.64	0.153
Weight (kg)	70.44 ± 11.64	80.76 ± 25.75	76.66 ± 33.20	0.286
Length (cm)	162.44 ± 4.65	162.26 ± 6.80	162.1 ± 7.30	0.512
Anesthesia time (min)	87.78 ± 20.23	80.76 ± 25.75	76.66 ± 33.20	0.146
Surgery time (min)	73.33 ± 21.42	65.84 ± 23.40	67.83 ± 32.12	0.272
ASA I/II	18/12	18/12	19/11	0.357

Values are presented as number and percentage.

Moreover, there was a significant difference between groups in terms of induction and maintenance of anesthesia and overall amount of consumed propofol and remifentanil. The amount of propofol used for induction was significantly higher in group S compared to groups PS and NS (P < 0.05), and the amount of propofol consumed by individuals in group PS was also significantly higher than in group NS (P < 0.05). Analysis of the total consumption of propofol showed that consumption in group S was significantly higher compared to groups PS and NS (P < 0.05), and the amount of propofol consumed by group PS was significantly higher than in group NS (P < 0.05). Furthermore, total remifentanil consumption by group S was significantly higher compared to group NS (P < 0.05) (Table 3).

**Table 3:** The consumption of propofol and remifentanil by group (mean ± SD).

	Induction propofol(mg)	Total propofol(mg)	Induction remifentanil (μg)	Total remifentanil(μg)
Group S	102.76 ± 12.97	179.38 ± 34.13	37.17 ± 6.95	1315.10 ± 381.63
Group PS	84.53 ± 16.97*#	150.50 ± 32.77*#	36.17 ± 7.73	1240.70 ± 492.97
Group NS	63.17 ± 17.77*	119.37 ± 40.78*	35.47 ± 6.61	1010.13 ± 417.05*

SD: Standard deviation. P-values compared among groups. *: P < 0.05 compared with group S #: P < 0.05 compared with group NS

**Figure 2:**
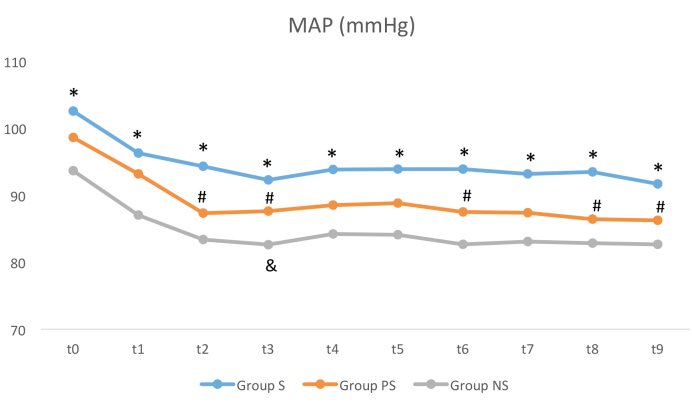
Mean arterial pressure (MAP) in groups. *Group S compared with group NS, # group S compared with group PS, and group PS compared with group NS.

Statistically significant changes were observed in the analysis of hemodynamic data, mean arterial pressure,and heart rate. The mean arterial blood pressure (MAP) in group S was higher than in group NS at all measurement times (t0–t9) (P < 0.05). Furthermore, MAP values of group S were significantly higher than those of group PS at t2, t3, t6, t8, and t9 (P < 0.05). Meanwhile, the MAP value of group PS was higher than that of group NS only at t3 (P < 0.05) (Figure 2).

**Figure 3:**
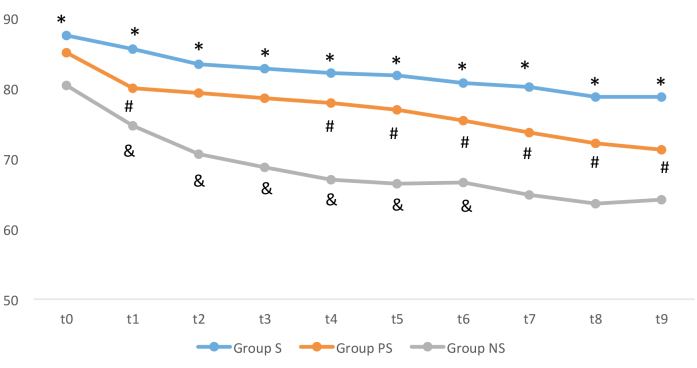
Patient heart rates (HRs) in groups.

Heart rate values in group S were higher than those of group NS at all time points (t0–t9) (P < 0.05). The heart rate values were also higher in group S compared to group PS at t1, and t4–t9 time points (P < 0.05). In addition, heart rate values in group PS were higher than those of group NS at t1–t6 time points (P < 0.05) (Figure 3).

## 4.Discussion

Smoking is a harmful habit that affects individuals and society by interfering with the treatment of chronic diseases and causing premature death. The results of our study show that while the anesthetic need in smokers was greater than in passive smokers and nonsmokers, passive smokers needed more anesthetic compared to nonsmokers. Nonsmokers who passively inhale cigarette smoke are also exposed to the damage caused by smoking.

There are a very limited number of studies that investigate anesthetic requirements for smokers. Lysakows et al. [4] reported that smokers required more propofol than nonsmokers. Similarly, in our study we determined that perioperative total propofol consumption in smokers was 50% greater compared to nonsmokers and 19% greater compared to passive smokers.

Cigarette smoke contains chemicals such as nicotine, carbon monoxide, nitrogen oxides, volatile aldehydes, hydrogen cyanide toxins, and polycyclic aromatic hydrocarbons (PAHs). Polycyclic aromatic hydrocarbons are the most important factor affecting the liver cytochrome P450 enzyme system (CYP) [5]. The cytochrome P450 enzyme system is the first defense mechanism against potentially harmful substances that the body encounters. In humans, approximately 30 CYP isoenzymes are responsible for drug metabolism; the most important of these are CYP3A4 and CYP2D6. Many drugs, including volatile anesthetic agents, are metabolized by CYP3A4 isoenzyme. However, cigarette smoke interacts with CYP1A1, CYP1A2, and CYP2E1 enzymes, allowing the hepatic effects of PAHs to manifest within 3–6 h and reach the maximum level within 24 h. Increased smoking leads to a proportional increase in enzyme induction [5]. CYP1A2 which metabolizes drugs such as theophylline, imipramine, paracetamol, and phenacetin is mainly localized in the liver and is induced by smoking. Smoking modifies enzyme activity and leads to an increase in the theophylline requirement in asthmatic patients and the haloperidol requirement in psychiatric patients. Polycyclic aromatic hydrocarbons and nicotine have also been reported to induce the CYP2E1 enzyme system [5].

UDP-glucuronyl transferase (UGT) is the major glycoprotein that resides in the membrane of endoplasmic reticulum. In addition to various environmental factors, smoking affects the activity of UGT. In humans, there are approximately 24 variants of UGT gene. The UGT 2B7 subset variant, which plays an important role in the metabolism of morphine and codeine, is induced by PAH in cigarette smoke. The major metabolites of morphine, morphine-3-glucuronide (M3G) and morphine-6- glucuronide (M6G), have 50 times more analgesic efficacy compared to morphine itself. Cigarette smoke induces UGT2B7 enzyme, and therefore increases the requirement for morphine by reducing the formation of M6G. Similar to morphine, smoking resulted in increased requirements for dextropropoxyphene and pentazocine in the perioperative and postoperative periods [5]. Glasson et al. reported that smoking changed the pain threshold. The same study also suggested that this change might have occurred via receptor-mediated UGT enzyme induction or by affecting liver clearance of morphine [6]. Rogers et al. [7] administered patients with a standard dose of 60 mg of codeine, and found that codeine clearance accelerated in smokers. The analgesic effect of codeine emerges by conversion to morphine via demethylation mediated by the CYP2D6 enzyme, and alternatively, through conversion to its active metabolite, codeine–6-glucuronide, as a result of demethylation by CYP3A4 enzyme. Morphine is primarily metabolized to normorphine via UGT and CYP3A4 enzymes [5]. In addition, Yue et al. [8] showed that smoking accelerates codeine glucuronidation without using O- and N-demethylation. Stanley et al. [9]. reported increased fentanyl consumption and an associated increase in the frequency of side effects such as rigidity and hypertension in smoking patients undergoing coronary artery bypass graft (CABG). Several investigators in the above studies have connected the observed increased opioid consumption in smokers with opioid liver metabolism. Although the remifentanil used in our study is metabolized independent of the liver, we saw that consumption was 30% greater in smokers than nonsmokers, which suggests that there might be different mechanisms for opioid requirements. In support of this view, nicotine has been reported to have antianalgesic effects, suggesting that it may enhance pain perception in patients. In vitro studies on neuronal physiology have shown that nicotine increases the transduction in sensory nerves. On the other hand, in vivo studies have indicated that nicotine has analgesic effects [10]. Pomerleau [11] reported increased tolerance to controlled pain stimulus (cold pressure response) in smokers. Rau et al. [12] tied the pain relief effect to nicotine and showed that the pain threshold associated with carotid baroreceptor stimulation increased proportionally with increasing doses of nicotine. However, the analgesic effect of nicotine is not fully understood. Furthermore, a person would have to have smoked for several years in order for it to affect pain tolerance; therefore, smoking alone does not explain the increase of intraoperative analgesia requirements [5].

Limitations of our study included a single-sex study group consisting of women only. In order to eliminate variability in anesthetic and analgesic requirements due to sex differences and surgery type, we limited our study to female patients undergoing the same type of surgery.

In conclusion, we determined that both active and passive smokers have higher anesthetic and analgesic requirements compared to nonsmokers; consequently, it is necessary to take precautions against possible complications.
